# Assessing Local Differential Privacy for Compliance with the Personal Data Protection Law in Integrated Data Systems

**DOI:** 10.1371/journal.pone.0342692

**Published:** 2026-03-30

**Authors:** Randa Aljably

**Affiliations:** College of Computer Science and Information Technology, Shaqra University, Shaqra, Saudi Arabia; MIT, UNITED STATES OF AMERICA

## Abstract

Organizations increasingly integrate and share person-level data across internal platforms and external partners to enable analytics, digital services, and evidence-based decision making. However, combining quasi-identifiers across systems and releases can enable re-identification via linkage attacks, creating regulatory compliance and trust risks. This paper proposes an operational methodology for (i) identifying direct identifiers and quasi-identifiers (QIs), (ii) quantifying baseline re-identification risk using uniqueness and prosecutor-style risk proxies, and (iii) applying Local Differential Privacy (LDP) to reduce link-ability prior to data sharing. We implement categorical LDP using a Generalized Randomized Response (GRR) mechanism and evaluate privacy–utility trade-offs through a sensitivity analysis over the privacy budget ε. Utility is quantified using (a) distributional distortion (total variation distance) and (b) downstream task performance (job-title classification). We further address reviewer concerns by discussing repeated releases, privacy accounting as mitigations for longitudinal deployments, and by improving figure readability and updating related work with recent studies.

## Introduction

Across sectors such as public administration, healthcare, education, and finance, organizations increasingly operate integrated data systems that consolidate records from multiple sources (e.g., HR, ERP, CRM, learning platforms, and external registries). Integration improves operational efficiency and enables cross-domain analytics, but it also increases privacy exposure: combinations of quasi-identifiers (QIs) can uniquely distinguish individuals, and attackers can exploit auxiliary datasets to link released records back to data subjects. [1–3]. Modern data-protection authorities therefore emphasize both (i) effective de-identification/anonymization and (ii) evidence that re-identification risk is sufficiently low in the intended sharing context. In Saudi Arabia, the Personal Data Protection Law (PDPL) frames anonymization as a process intended to prevent re-identification and expects controllers to apply safeguards and demonstrate their effectiveness. Similar expectations appear internationally, although explicit technical thresholds are often not prescribed [3]. Table 1 outlines key terms and concepts as defined in the PDPL.

**Table 1:** PDPL privacy concepts and operational interpretation.

Indicative requirement	Operational interpretation for sharing	How this study addresses it
Identify regulated data (personal and sensitive categories)	Determine which fields can identify individuals directly/indirectly and which are high-risk (sensitive).	We separate direct identifiers from QIs and flag sensitive attributes for stricter handling.
Demonstrate effective anonymization (re-identification should be infeasible under plausible attacker knowledge)	Anonymization must be evidenced by measurable reduction in linkage/ReID risk.	We quantify baseline ReID risk (uniqueness, prosecutor-style risk) and evaluate reduction after LDP.
Justify processing and balance against privacy risk	Processing must be justified; higher-risk sharing requires stronger safeguards.	We report privacy–utility trade-offs across ε to support evidence-based balancing decisions.
Reassess risk when processing purpose changes	New purposes can change risk and attacker assumptions; reassessment is required.	We treat QI selection and threat model as scenario-dependent and recommend re-running risk assessment when purpose changes.
Account for third-party collection/integration (auxiliary data increases linkage risk)	Integration increases attacker power by providing auxiliary information for linkage.	We assume plausible auxiliary data and evaluate linkage risk using QI-based attacker models and exact-match proxies.
Apply and evidence security safeguards (privacy protection is operational, not only legal)	Controls must be implemented and auditable, including for repeated sharing over time.	We recommend governance controls: privacy budget ledger, bounded releases, and memoization/permanent RR for repeated releases.

Two operational challenges arise. First, organizations need a repeatable risk-assessment workflow that translates regulatory anonymization requirements into measurable privacy indicators under plausible attacker knowledge models. Second, when data are shared publicly or under weak trust assumptions, privacy mechanisms must remain robust against linkage attacks even if adversaries hold auxiliary datasets [4]. Local Differential Privacy (LDP) is a compelling option in such settings because it perturbs data at the individual level before release and provides a formal privacy guarantee controlled by a privacy budget.

This research is expected to provide value for organizations seeking to ensure that their anonymization practices align with the PDPL’s standards, particularly for data collected through internal and external system integration.

**Fig 1:**
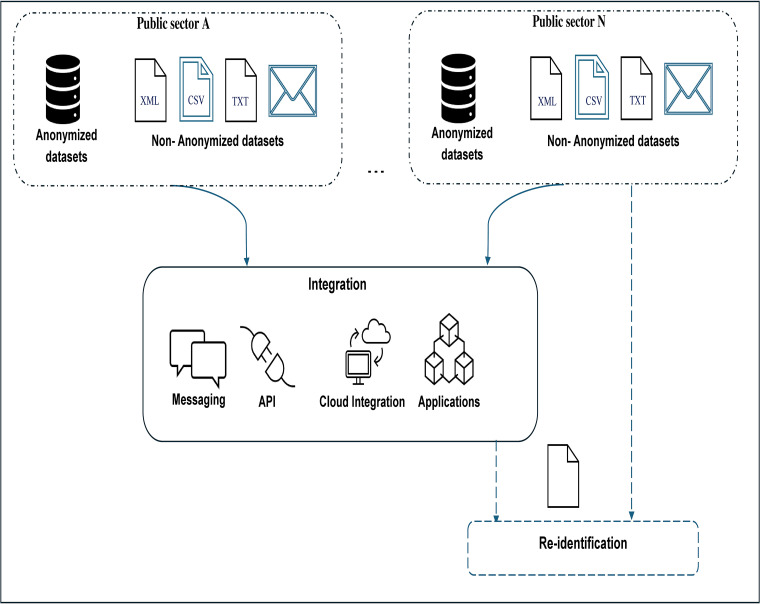
Linkage-based re-identification risk in integrated data systems. The figure illustrates how quasi-identifiers from multiple organizational systems can be combined by an adversary to re-identify individuals. It highlights the increased privacy exposure caused by cross-system data integration and motivates the need for risk assessment in integrated environments.

The research paper evaluates the proposed method for re-identification risks (ReID risk) against linkage threats. Linkage threats are characterized by using complementary data or background information to trace anonymized datasets back to individuals [5]. In this case, adversaries attempt to re-identify data subjects in anonymized datasets by using non-anonymized datasets available in public sector agencies or open data portals [6]. Fig 1 provides a visualization of the problem. This paper applies the evaluation approach to different data types, including employee personal data and auxiliary data, to assess anonymization effectiveness. The approach aims to contribute to the ongoing discussion on how anonymization methods can be used to comply with evolving data protection regulations [3].

This research paper contributes to the literature by investigating the following research questions:

RQ1: What is the appropriate methodology for public sector organizations to assess the risk of subject re-identification and determine the de-identification target and level for protecting employee privacy?

RQ2: Can the application of LDP to integrated organizational data eliminate the estimated probability of re-identification, achieve PDPL compliance, and preserve personal data privacy?

This paper’s proposed approach is not straightforward because the organizational data under study is collected through internal and external integration with other public sector organizations, resulting in large data repositories. Since there are no clear guidelines on storing these data repositories, this aggregation method impacts data protection. Therefore, privacy concerns are amplified, as privacy breaches—whether intentional or accidental, manual or electronic—require stringent governance.

The remainder of this paper is structured as follows: **Preliminaries** provide background concepts on anonymization techniques. **Literature Review** summarizes related work. **Methodology** describes the proposed approach and experimental setup, followed by the results and discussion. Finally, the **Conclusion** highlights key findings and future directions.

### Preliminaries

Organizations should use appropriate data anonymization techniques to prevent security breaches targeting employee personal data. These attacks re-identify their records in shared datasets through aggregate releases or open data linkage. However, these techniques vary in terms of generalization, suppression, k-anonymization, and limitations [7].

When adding noise to the data, the approach differs depending on the context. In the case of sharing data with a trusted party, noise can be added to the aggregates (globally) before aggregation. In contrast, sharing data publicly requires adding noise directly to individual data points (locally).

### Local Differential Privacy (LDP)

The approach on which Differential Privacy (DP) is based involves selectively including or excluding randomly selected individual records from the dataset. To illustrate, suppose that the concepts D and D’ represent neighboring datasets that differ by exactly one record. In eq.1 For all D and D’, there exists a mechanism M that adds randomly generated noise derived from a probability distribution, such as the Laplace distribution. As shown in eq.2, the output O of the Laplacian mechanism M, depicted in eq.3, is similar for all D and D’, depending on the privacy budget ∊ ∈ [0, 1] and noise scale SC, which is proportional to the sensitivity or the maximum change in the output after inclusion or exclusion [7].


$$Pr\left[\;M(D)\in O\right]\leq \;e^{\epsilon \;}Pr[M\left(D^{\prime }\in O\right)]$$ (1)

$$Lap_{M}(D,Sc)=r(d)+Lap(Sc)$$ (2)

$$Lap(Sc)=\;\frac{Sensitivity}{\epsilon }$$ (3)

Many controllers have considered various anonymization techniques to solve data protection and privacy issues. However, although this assumption might award the controller legal compliance with their internal data protection requirements, it may obscure actual data usage or, in the worst-case scenario, render the controller non-compliant with national laws. In response to this challenge, this paper focuses on the need for anonymization that is successfully used for sharing organizational private data in external integrations. Therefore, this research’s main objective is to achieve the preservation of organizational private data while maintaining usability and allowing proper analysis or reporting on statistics and decision-making.

### GRR for categorical attributes

For a categorical domain of size d, Generalized Randomized Response (GRR) reports the true value with probability


$$p=\frac{e^{\varepsilon }}{e^{\varepsilon }+d-1},$$

and reports any other value uniformly with probability (1−p)/(d−1). GRR is appropriate for categorical attributes and is widely used in LDP deployments and analyses.

Another problem identified in the literature is that several known anonymization algorithms are not sufficient to accomplish the privacy preservation level mandated by data protection laws such as the General Data Protection Regulation (GDPR), the General Personal Data Protection Law (LGPD) [8]. These methods are especially insufficient when data is aggregated from multiple sources. In data aggregation, some of the datasets are anonymized without the identification of data subjects. Unfortunately, those subjects can be easily identified by linkage to other non-anonymized datasets or publicly available data (e.g., found on webpages) that are aggregated from other sources, such as other public sectors in integration initiatives. [9].

The rationale for selecting LDP as an anonymization method stems from the need to counterbalance the advantages and risks of using anonymization as a form of compliance. This paper advances the hypothesis that while anonymization is an important tool developed by compliance-oriented governance to increase data protection, anonymization should not hinder the intended learning or reporting process of the underlying data. In addition, it should survive the Membership Inference Attack (MIA) used to match anonymized to non-anonymized records in different datasets.

### Literature review

With the worldwide enactment and implementation of regulations for data protection laws, privacy toolkits for Big Data (BD) have increasingly gained attention to protect collectors’ private data. The underlying principle of these tools is the removal of Personally Identifying Information (PII) from logs and repositories without impeding the intended use of BD or preventing re-identification when needed [4]. The most prominent anonymization algorithms used to ensure compliance with data protection regulations such as the GDPR, the LGPD [10,11], and PDPL include k-anonymization and extensions such as l-diversity and t-closeness, δ-presence, δ-disclosure privacy [12], and Bayesian statistics [4]

The k-anonymization algorithm uses generalization or suppression techniques, but it does not produce promising results on high-dimensional datasets [13]. By contrast, l-diversity is significantly more challenging to implement yet it cannot guarantee absolute privacy protection for anonymized BD [4]. In k-anonymization, the values of the quasi-identifiers in an anonymity data cell are exuberated to reach the least k records with identical identifier values. Then the simplest form of l-diversity requires that for any quasi-identifier, there exists at least l distinct value in the cell [14]. Whereas for t-closeness, the difference between the distribution in the whole sample and the distribution of the l values does not exceed the threshold t. For each of these algorithms, k-anonymization, l-diversity, and t-closeness, the literature indicates that the resulting anonymized datasets are insufficient for performing their original tasks, such as anomaly detection in log records [12,15]. According to [4], the t-closeness algorithm outperformed the other two algorithms in minimizing information loss.

The δ-disclosure algorithm shares the same enhancement as the k-anonymization algorithm in overcoming the correlation between the number of attributes in a dataset and the probability of information loss. Similarly, the Bayesian statistical approach adds random Gaussian noise and known distributional properties to a random selection of variables (categorical and continuous) in a released dataset, thereby taking into account the availability of both identifying and sensitive variables for data subjects that adversaries can use for de-anonymization [12].

The National Institute of Standards and Technology (NIST) acknowledged Data Protection (DP) in their data anonymization framework for government data sharing [7]. DP is preferred by governmental and semi-governmental organizations as it does not require assumptions about the auxiliary parameters, regardless of the number of dataset releases and an attacker’s computational power. Additionally, DP achieves similar results on different types of data.

Data types include peer and location information, as found in Geographical Location Oriented Network (GLON) [3], IP addresses [3], images and videos [5,16], medical records, metadata, various governance documents in multiple file formats (PDF, XML, and DOCX) [15], analytical systems and data lakes [10], and cloud event logs [11]. Other approaches [5,16] have concluded that no optimal anonymization software exists for achieving data privacy compliance with GDPR while maintaining data utilization. Therefore, such approaches have developed policies and open-source libraries to effectively identify and handle sensitive information used for research, education, and prosecution [17]. Additionally, the authors in [16] reported a proposed approach using holistic deep learning evaluation of anonymized image pairs, where the model assesses the cumulative image instead of focusing on anonymized subjects.

GDPR defines re-identification as the use of additional information (also known as auxiliary information) leading to the identification of individuals [16]. This de-anonymization process estimates the background information by correlations and data cross-referencing [15] for each anonymized subject, then uses it to infer the identity of that subject causing a privacy breach. The literature demonstrates two cases where a non-anonymized DS can infer to an anonymized one: first, in batch linking, where data about the same DSs are correlated to various sources in advance; and second, ad hoc linking, where data about the same DSs are correlated in real-time [10].

Batch linking was explored alongside k-anonymization in [18], where the results indicated the lowest ReID risk for unbiased samples in the dataset’s population. By contrast, the Bayesian method based on ad hoc linking performed remarkably well as the disclosure risks were insensitive to correlations between the identifiers.

## Methodology

As departments within an organization share employee data internally and externally (within and across sectors), it is crucial to identify and follow an approach to quantify the risk of re-identification. Furthermore, it is necessary to manage ReID risk by determining the appropriate level of de-identification depending on the context of the data release [19].

The following sections provide an algorithm description of Algorithm 1 and outline the recommended steps that organizations should follow to quantify the probability of re-identification.

**Algorithm 1. Re-identification algorithm.**

***BEGIN Re-Identification Algorithm***

*//* ***Step 1****: Determine the format of data sharing between parties*

*DetermineFormatOfDataSharing()*

*//* ***Step 2:*** *Determine the identifiers in the data set*

*IdentifyIdentifiersInDataSet()*

*//* ***Step 3:*** *Start iterating over the data*

*FOR each record in dataset DO*

*//* ***Step 4****: Calculate the attempt risk for the current record*

*attemptRisk = CalculateAttemptRisk(record)*

*//* ***Step 5:*** *Calculate the probability of re-identification for the subject in the sample*

*reIdentificationProbability= CalculateReIdentificationProbability(record)*

*//* ***Step 6:*** *Calculate the re-identification risk for subjects in the entire population (if multiple releases)*

*populationRisk = CalculatePopulationReIdentificationRisk(record)*

*//* ***Step 7:*** *Calculate the overall risk for the population dataset*

*overallRisk = CalculateOverallRisk(populationDataset)*

*//* ***Step 8:*** *Measure the uniqueness of the current record*

*uniqueness = MeasureUniqueness(record)*

*//* ***Step 9:*** *Compare the uniqueness against the risk threshold*

***IF*** *uniqueness > acceptableUniquenessThreshold THEN*

*//* ***Step 10:*** *Apply an anonymization algorithm to reduce uniqueness*

*AnonymizeData(record)*

***ELSE***

*//* ***Step 11:*** *Check if dataset meets privacy requirements*

***IF*** *uniqueness <= acceptableUniquenessThreshold THEN*

*//* ***Step 12:*** *The dataset is in accordance with privacy requirements*

***PRINT “Dataset meets privacy requirements.”***

***END IF***

***END IF***

***END FOR***

***END Re-Identification Algorithm***

### Find identifiers in the data

Certain variables can directly or indirectly identify subjects in a dataset. According to GDPR, NIST, and the California Consumer Privacy Act (CCPA), these identifiers are categorized into two groups: direct identifiers, which can explicitly reveal an individual’s identity in the dataset. In an employee dataset called EMP-DS, these variables are typically as follows: Full name, Employee ID, National ID number, Date of birth, Home address, IP address or Email address, Contact number, and Biometric data. When Employee ID is pseudonymized, the rest of the variables are assumed to have no analytical value and can be removed from the dataset. On the other hand, indirect (quasi) identifiers need to be combined with other information to identify an individual in the dataset. Indirect identifies include Job title, Department or Location, Birth date, Hire and termination dates, Financial data, Gender, Religion, and Education level. The indirect identifiers are the ones used in calculating the risk of re-identification, as demonstrated in the upcoming practical example.

The main variables included in the EMP-DS dataset fall under the Personal Data category, for example, Full name and Identification numbers. Auxiliary data, which supports the main data, including Job title, Rank, Job group, Job family, and Academic qualification details. Table 2. shows an overview of the dataset with the main variables, and Table 3 shows the data model for the dataset.

**Table 2:** Employee Dataset (EMP-DS) attributes and categories.

Personally identifiable information (PII) and Auxiliary Data (AD)	Column name	Data type
PII	Arabic First Name	string
PII	Arabic Second Name	string
PII	Arabic Last Name	string
PII	English First Name	string
PII	English Second Name	string
PII	English Last Name	string
PII	Employee Number	int
PII	Identity Number	int
AD	Birthplace	location
AD	Gregorian Birth Date	date
PII	Passport Number	int
PII	Original Home Number	int
AD	Is Male Gender	Boolean
AD	Nationality Code	lookup
AD	Marital Status Code	lookup
AD	Religion Code	lookup
PII	Postal Address	lookup
AD	City Code	lookup
AD	Zip Code	lookup
PII	Email Address	string
PII	Home Telephone Number	int
AD	Work Telephone Number	int
AD	Work Telephone Extension	int
PII	Mobile Number	int
AD	Is Special Needs	Boolean
AD	Special Need Code	lookup

**Table 3:** Employee Dataset (EMP-DS) data model.

EMP-DS	
PK	IDENTITY_NUMBER INTEGER (10) ARABIC_FIRST NAME VARCHAR (15) ARABIC_SECOND_NAME VARCHAR (15) ARABIC_LAST_NAME VARCHAR (15) ENGLISH_FIRST_NAME VARCHAR (15) ENGLISH_SECOND_NAME VARCHAR (15) ENGLISH_LAST_NAME VARCHAR (15) BIRTHPLACE LOCATION GREGORIAN_BIRTH_DATE DATE PASSPORT_NUMBER INTEGER (10) ORIGINAL_HOME_NUMBER INTEGER (10) IS_MALE_GENDER BOOLEAN NATIONALITY_CODE LOOKUP MARITAL_STATUS_CODE LOOKUP RELIGION_CODE LOOKUP POSTAL_ADDRESS LOOKUP CITY_CODE LOOKUP ZIP_CODE LOOKUP EMAIL_ADDRESS VARCHAR (15) HOME_TELEPHONE_NUMBER INTEGER (10) WORK_TELEPHONE_NUMBER INTEGER (10) WORK_TELEPHON_ EXTENTION INTEGER (10) MOBILE_NUMBER INTEGER (10) IS_SPECIAL_NEEDS BOOLEAN SPECIAL_NEED_CODE LOOKUP

### Calculate the attempt risk

Calculating the attempt risk is critical when data is exchanged according to a sharing agreement with third parties, under the following conditions: the recipient is a known organization, the agreement prohibits attempts at re-identification; and the agreement mandates compliance with local laws and regulations specifying privacy controls and auditing requirements.

### Calculate the attempt risk

Calculating the attempt risk is critical when data is exchanged according to a sharing agreement with third parties, under the following conditions: the recipient is a known organization, the agreement prohibits attempts at re-identification; and the agreement mandates compliance with local laws and regulations specifying privacy controls and auditing requirements.

The probability of an attempt must be calculated whether the attempt to re-identify the data is deliberate, accidental, or a result of a security attack or data loss (breach). This probability, known as the attempt risk, can be calculated as follows [20]:


Pro(attempt) = max { Pro(deliberate), Pro(accidental), Pro(breach)}. (4)

### For each subject, use indirect variables to calculate the probability of subject re-identification in the sample population

For example, in the EMP-DS dataset used in this research, the total number of registered employees is approximately 2,100; however, in this scenario, as the risk of identification arises of linkage threat to 2000 records present in open datasets, we will address these records. The data fields published in open datasets include Gender, Educational rank (special for educational staff), Job title, Functional group, Functional family, Qualification university country, and University name. Amongst these, Job title and Gender were selected. Subjects that have the same indirect variable values in the sample are sorted into the same class, and the probability of the ReID risk is calculated as (1/ class-instance).

Table 4 shows an example of the dataset sample, along with equivalence instances in each class and each individual ReID risk.

**Table 4:** Class instance and the ReID risk in Employee Dataset (EMP-DS) sample.

Employee No.	Job title	Gender	Class-instance	ReID Risk
UE-001	Teaching assistant	F	A (2)	0.50
UE-002	Lecturer	F	B (4)	0.25
UE-003	Lecturer	F	B (4)	0.25
UE-004	Lecturer	F	B (4)	0.25
UE-005	Assistant Professor	F	C (1)	1
UE-006	Teaching assistant	F	A (2)	0.5
UE-007	Lecturer	F	B (4)	0.25
UE-008	Assistant Professor	M	D (2)	0.50
UE-000	Assistant Professor	M	D (2)	0.50
UE-010	Teaching assistant	M	E (1)	1

### Calculate ReID risk for subjects in the entire population

**Fig 2:**
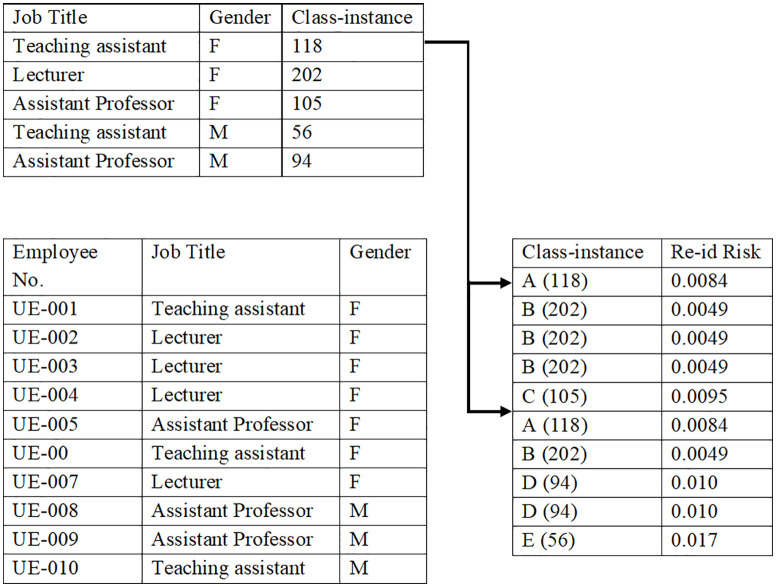
Record-level re-identification risk in the employee dataset (EMP-DS). Each bar represents the calculated prosecutor-style re-identification risk for an individual record based on indirect identifiers (Job Title and Gender). Higher values indicate greater vulnerability to linkage attacks.Calculate The Overall Risk for The Population Dataset.

The database sample of subjects contained 817 records [6], with 118 females classified as teaching assistants. Therefore, for example, the ReID risk for UE-001 is 1/118 = 0.0084. Since the population size is moderate, the individual risks for each record are acceptable; however, if the sample size is smaller, the risks will exceed the identification threshold and label the data risky [20]. The individual subject (record level) ReID risk is shown in Fig 2.

Two approaches are used to calculate the overall risk: Average and Maximum. Maximum risk is calculated as max(∑individual risks) in the dataset, while average risk is calculated as $\frac{\sum individual\;risks)}{dataset\;recors}$ across the dataset. Therefore, the risk in the sample is calculated as follows:


$${Pro}_{max}(re-id\;|attempt)=\text{max}(0.50,\;0.25,\;0.25,\;0.25,1,0.5,\;0.25,\;0.50,\;0.50,\;1)=1$$

$${Pro}_{avg}(re-id\;|attempt)=\frac{\begin{array}{c} (0.50+\;0.25+\;0.25+\;0.25+1+0.5+\;0.25+\;0.50\\ +0.50+\;1) \end{array}}{10}=0.5$$

While the risk across the entire population is calculated as follows:


$${Pro}_{max}(re-id\;|attempt)=\text{max}(\text{0}.\text{0084},\text{ 0}.\text{0049},\text{ 0}.\text{0049},\text{ 0}.\text{0049},\text{0}.\text{0095},\text{0}.\text{0084},\text{ 0}.\text{0049},\text{ 0}.\text{010},\text{ 0}.\text{010},\text{ 0}.\text{017})=\text{0}.\text{017}$$

$${Pro}_{avg}(re-id\;|attempt)=\frac{\begin{array}{c} (\text{0}.\text{0084},\text{ 0}.\text{0049},\text{ 0}.\text{0049},\text{ 0}.\text{0049},\text{0}.\text{0095},\text{0}.\text{0084},\text{ 0}.\text{0049},\\ \text{ 0}.\text{010},\text{ 0}.\text{010},\text{ 0}.\text{017}) \end{array}}{10}=0.00829$$

As the preceding calculations indicate, the maximum risk is substantially narrower than the average risk. Therefore, it is used in case of open data sharing where, as the risk of re-identification of private data records using publicly available data is high, the records with the highest probability of re-identification are the most likely targets of the attacks. Meanwhile, either maximum or average risk can be used as the threshold to achieve secure data sharing [20,21].

### Measure the uniqueness

Despite obtaining a low ${Pro}_{avg}(re-id\;|attempt)$ in the EMP-DS dataset, in the case of sharing sensitive personal data, ${Pro}_{avg}(re-id\;|attempt)$ may not be sufficient as the sole measure to allow sharing the dataset without further consideration.

Therefore, as an additional measurement for de-identification, organizations should calculate the percentage of unique records and show which records can be safely grouped in an equivalence class, as compared to complementary unique records that require more processing. This approach is then added to the previous set of metrics as follows:


$${Pro}_{max}(re-id)={Pro}_{max}(re-id\;|attempt)\;\times Pro(attempt)$$ (5)

And


$${Pro}_{avg}(re-id)={Pro}_{avg}(re-id\;|attempt)\;\times Pro(attempt)$$ (6)

As shown in Table 5.

**Table 5:** Maximum and average reidentification probability and uniqueness.

Context	${Pro}_{max}\left(re-id\right)$	${Pro}_{avg}\left(re-id\right)$	% unique records
Sample Dataset	1	0.5	20%
Entire Population	0.17	0.00829	0%

### Risk and utility metrics

Uniqueness (in Eq. 7). For dataset size Nand a QI set, let u be the number of records whose QI tuple occurs exactly once (equivalence class size = 1).


$$Uniqueness=\frac{\%\;unique\;records}{Acceptable\;value}$$ (7)

Prosecutor-style risk proxy. For record i, define $r_{i}=\text{1}/eq\_size_{i}$. We report the maximum and mean risk across records.

k-violation rate (evaluation threshold). For a threshold k, report the fraction of records with eq_size<k. We use k∈{2,5,10}as evaluation thresholds to contextualize baseline linkability (not as a post-LDP k-anonymity guarantee).

Statistical utility (distributional distortion). For each perturbed categorical feature, compute Total Variation (TV) distance between empirical distributions pre- and post-LDP. We also report on an average TV across the feature set.

Task utility (ML). Train a classifier to predict Job Title using selected features. Report accuracy and weighted F1 using identical train/test splits on raw vs LDP-perturbed features.

### Compare against the risk threshold

The threshold chosen by the controller or data custodian plays a crucial role as an evaluation criterion for anonymization algorithms and an optimization criterion for de-identification. It is necessary that its value balances the protection of records against de-identification and managing the utility and usefulness of the data.

The process for selecting the threshold considers

multiple factors, including privacy’s impact on sensitivity and possible harm. According to the literature, various approaches start from 0.5 for low risk and decrease to 0.05 for higher risk [14,22,23].

Some approaches use the threshold as an indicator of a statistical information deviation between a past record and a current one. For example, the authors in [22] set the value to 0.8. Another approach set the maximum probability of re-identifying a record to 0.5 for a dataset with zero population uniqueness. The number is noticeably higher than the commonly recommended threshold value of 0.09, whether in medical records or records holding personal information [22]. The Health Insurance Portability and Accountability Act (HIPAA) Privacy Rule set the threshold for re-identification to 0.33 when the dataset is shared with a trusted party and a probability between 0.09 and <0.05 when the dataset is publicly released [14].

In this paper, 0.09 is selected as the threshold for sharing data between organizations. The rationale for this threshold value is that it strikes a balance between testing ReID risk and maintaining data usability. As the data is shared with a governmental sector, the threshold can be applied to either max or average risk depending on safety requirements. In this scenario, it will be applied to the average.

Where:


$${Pro}_{max}*\;=\;\frac{{Pro}_{max}(re-id)}{Threshold}$$ (8)

$${Pro}_{avg}*\;=\;\frac{{Pro}_{avg}(re-id)}{Threshold}$$ (9)

As demonstrated in Table 6, if the shared dataset is substantially smaller than the population, or if the calculated ReID risk is less than the designated threshold, the result will not comply with privacy requirements. In this case, the dataset should be manipulated further to solve the problem.

**Table 6:** Comparison of uniqueness thresholds.

Size	Attempt Prob.	${Pro}_{max}$*	${Pro}_{avg}$*	Uniqueness	Result
Sample Dataset	0.1	$\frac{\text{1}}{\text{0}.\text{09}}$	$\frac{\text{0}.\text{5}}{0.09}$	$\frac{\text{20}\%}{\text{1}\%}$	Not Sufficient
Entire Population	0.1	$\frac{\text{0}.\text{17}}{\text{0}.\text{09}}$	$\frac{\text{0}.\text{00829}}{\text{0}.\text{09}}$	$\frac{\text{0}\%}{\text{1}\%}$	Sufficient

### Apply anonymization to non-compliant sets

**Fig 3:**
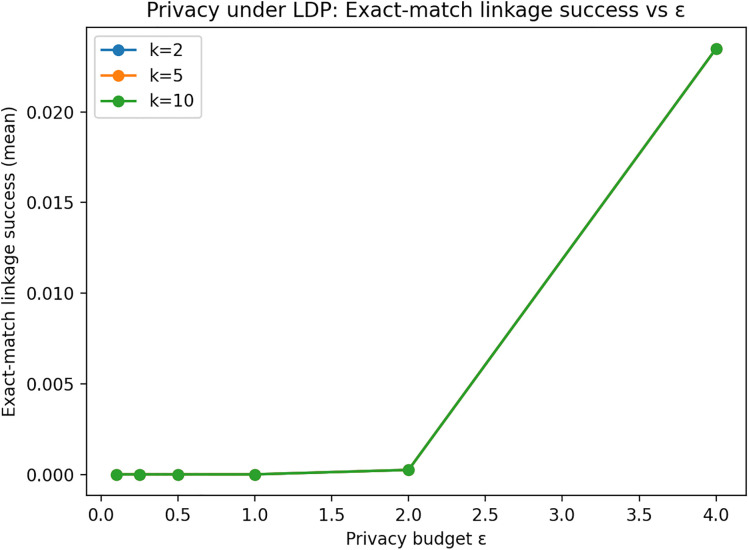
Exact-match linkage success under Local Differential Privacy. The figure shows exact-match linkage success rates for varying privacy budgets (ε). Lower ε values provide stronger privacy protection and substantially reduce the attacker’s ability to link records.

To solve the non-compliance issue with a dataset of sample size, Local Differential Privacy (LDP) is introduced. This technique is selected because LDP is immune to linkage attacks, even if an adversary has access to non-anonymized records. Therefore, access to non-anonymized records in open datasets would no longer permit a linkage attack to data subjects whose identifiers may or may not be present in the anonymized dataset [24]. Fig 3 shows the uniqueness levels of the sample dataset after applying LDP with a privacy budget of ∊ = 0.5 and k = 2 anonymity.

### Baseline linkability (pre-LDP)

Using the selected QI set, EMP-DS exhibits substantial link-ability. For example, at k=2(unique records), the baseline uniqueness is high (Uniq_raw = 0.7426, i.e., ~ 74.3% of records are unique on the QI tuple), and the baseline prosecutor-style average risk is pravg_raw = 0.8395 (Table 2). This motivates applying additional protection prior to sharing in weak-trust scenarios.

### LDP parameterization and sensitivity analysis

Local Differential Privacy (LDP) is governed by the privacy budget ε, where smaller ε increases randomization (stronger privacy) and larger ε preserves more signal (higher utility) [25]. Accordingly, we treat ε as the primary privacy–utility control and evaluate outcomes across a range of ε values rather than relying on a single setting. To contextualize baseline link-ability under different indistinguishability expectations, we also report metrics under k thresholds (k ∈ {2, 5, 10}), where k is used only as an evaluation lens (fraction with equivalence-class size < k) and not as a post-LDP k-anonymity guarantee.

**Fig 4:**
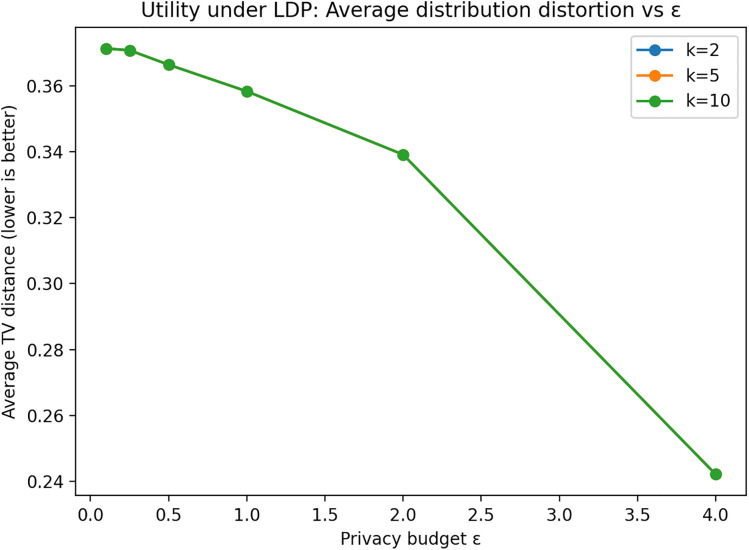
Distributional distortion under Local Differential Privacy. Average Total Variation (TV) distance between raw and LDP-perturbed attribute distributions across different ε settings. Lower distances indicate higher statistical utility.

Experiments are conducted on EMP-DS (n = 2000) using quasi-identifiers (QIs) {Gender, Qualification, University Country, University Name}. We apply Generalized Randomized Response (GRR) to categorical attributes and sweep ε ∈ {0.1, 0.25, 0.5, 1, 2, 4}, reporting averages over multiple random seeds. Privacy is evaluated using baseline link-ability on true QIs (uniqueness and prosecutor-style 1/eq_size risk) and an operational exact-match linkage proxy. As shown in Fig 3, decreasing ε sharply reduces linkage success: the exact-match attacker’s success is effectively zero for ε ≤ 1 and remains low even at higher ε (e.g., increasing only modestly at ε = 4), confirming that LDP substantially disrupts QI-based linkage. Utility is assessed via both distribution preservation and downstream task performance. Fig 4 shows that distributional distortion (average TV distance) decreases as ε increases, illustrating the expected privacy–utility trade-off. For task utility, we train a classifier to predict Job Title from {Gender, Qualification, Job Group, University Country, University Name}, perturbing only the features (labels are not perturbed). decreases as ε increases, illustrating the expected privacy–utility trade-off.

**Fig 5:**
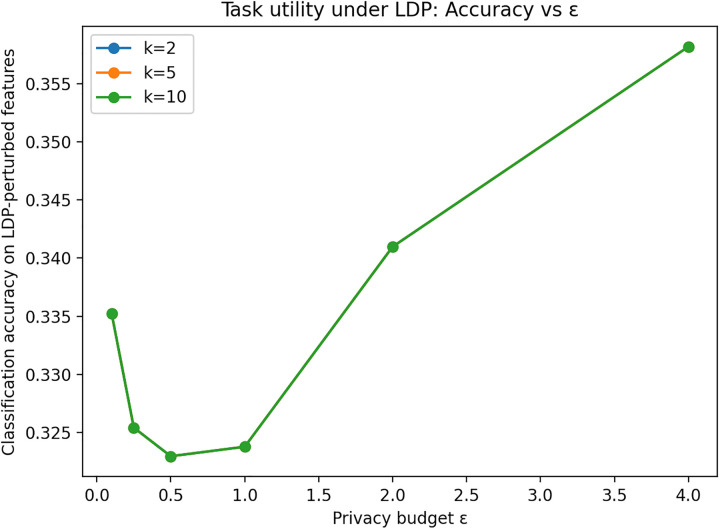
Classification accuracy under LDP-perturbed features. Classification accuracy for predicting Job Title using LDP-perturbed features. Accuracy improves as ε increases, reflecting the privacy–utility trade-off.

**Fig 6:**
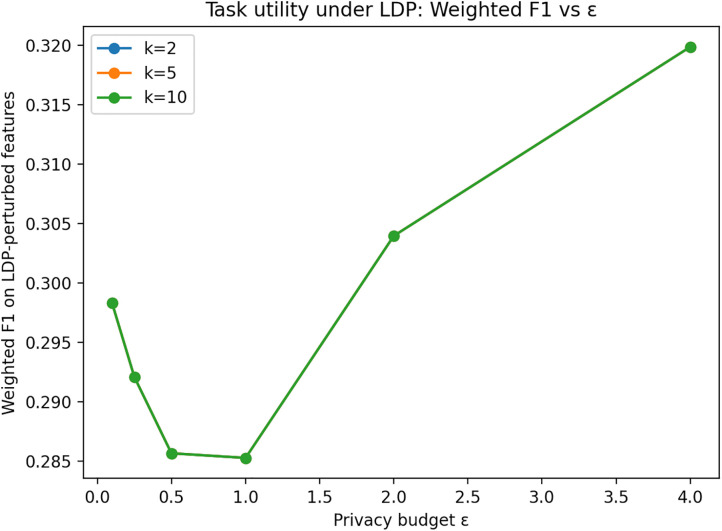
Weighted F1-score under LDP-perturbed features. Weighted F1-score for Job Title prediction across different LDP privacy budgets. Larger ε values improve predictive performance while maintaining privacy guarantees.

For task utility, we train a classifier to predict Job Title from {Gender, Qualification, Job Group, University Country, University Name}, perturbing only the features (labels are not perturbed). Fig 5 and Fig 6 indicate that classification accuracy and weighted F1 on LDP-perturbed features remain comparable to the raw baseline and generally improve with larger ε, demonstrating that useful predictive performance can be retained while substantially reducing linkability.

Overall, the sensitivity analysis confirms the expected trade-off: smaller ε strengthens privacy (lower linkage success), whereas larger ε improves statistical and task utility.

### Repeated releases, dynamic updates, and privacy accounting under LDP

In practical deployments, organizations rarely publish data once; instead, they often issue periodic releases (e.g., monthly/quarterly reporting) or share updated records over time [1,2]. Although LDP provides a formal guarantee per release, repeated releases increase the adversary’s inference opportunities, because the attacker can observe multiple independently perturbed outputs and exploit them jointly. Therefore, LDP in real systems must be treated as a budgeted longitudinal process, where privacy loss can accumulate across multiple releases under standard composition principles, motivating explicit privacy accounting and governance of cumulative privacy use [26].

To mitigate these risks, we recommend operational controls consistent with established LDP deployments: (i) allocate a per-release ε and maintain a privacy budget ledger to track cumulative use; (ii) apply memoization/ permanent randomized response when the same attribute is collected repeatedly (so repeated collection does not provide fresh independent observations that can be averaged over time); and (iii) enforce bounded release frequency and periodic review of ε allocation based on the sensitivity of attributes and intended use [26]. In addition, for evolving/longitudinal settings where user values may change over time, specialized LDP protocols have been proposed to reduce longitudinal privacy leakage while maintaining utility, supporting the need to explicitly address updates and repeated collection in the design.

**Fig 7:**
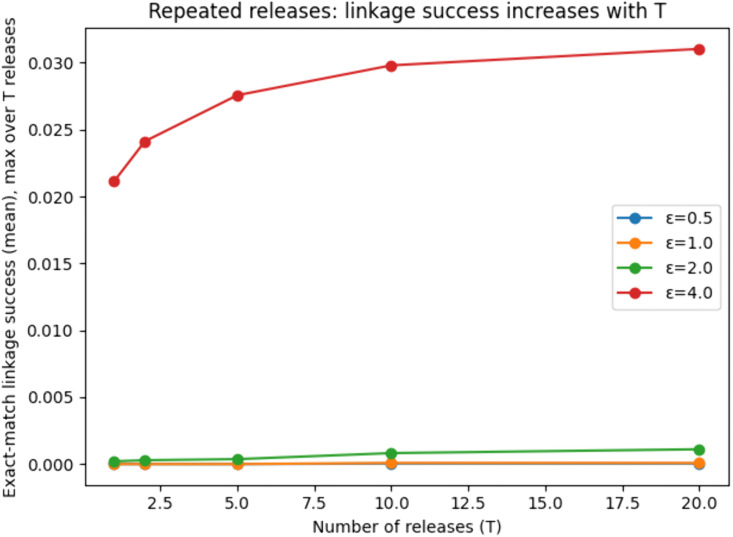
Effect of repeated data releases on linkage success. The figure shows the attacker’s exact-match linkage success under repeated data releases with fixed ε. Linkage success increases as the number of releases grows, demonstrating cumulative privacy loss.

To empirically illustrate repeated-sharing risk in our context, we simulated T releases of the same underlying dataset under fixed ε and varied T ∈ {1, 2, 5, 10, 20}. As shown in Fig 7, the exact-match linkage success increases with T (computed as the maximum success over the T releases to represent an attacker selecting the most favorable release), confirming that repeated releases create additional linkage opportunities even when each individual release is locally privatized.

**Fig 8:**
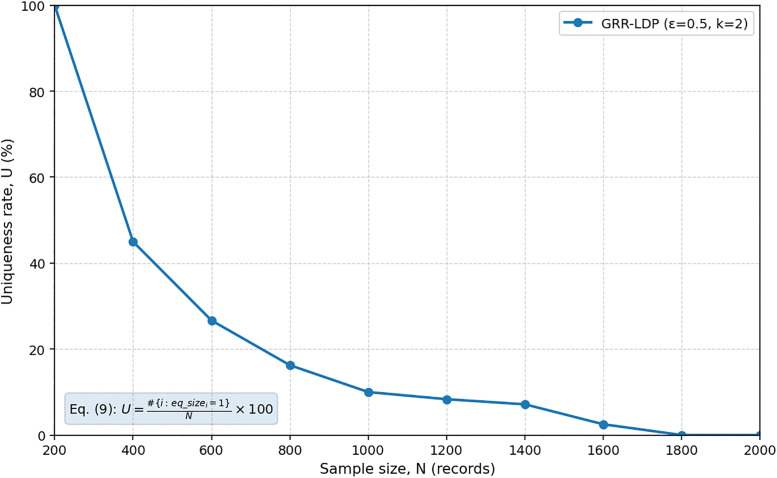
Reduction of uniqueness after applying Local Differential Privacy. Uniqueness levels in the sample dataset before and after applying LDP. The results show that LDP significantly reduces linkability and supports PDPL compliance.

As shown in Fig 8, the risk of re-identification based on population uniqueness for the sample dataset can be reduced using LDP until it reaches a sufficient level. In this way, the research questions are addressed, and the above-mentioned steps could be adopted within an organization’s compliance and assurance operations to promote satisfying PDPL data subject privacy requirements. With the research context in mind, it is noteworthy that the proposed method provides value, particularly for data exchange through systems integration, enabling the organization to attain compliance with the PDPL privacy mandates using the suggested level of LDP anonymization.

## Results and discussion

A closely related study is Arcolezi et al. [18], which investigates privacy threats (re-identification and attribute inference) against LDP protocols for multidimensional data, evaluating several widely used mechanisms (e.g., GRR, RAPPOR, hashing/unary encoding variants) and proposing a countermeasure to improve robustness while maintaining utility. Our work differs in scope and contribution: rather than focusing on attacking/benchmarking multidimensional frequency-estimation protocols, we propose an audit-friendly, compliance-oriented methodology for integrated tabular systems, combining ReID-risk assessment, LDP anonymization, and privacy–utility evaluation aligned with organizational PDPL needs. Table 7 demonstrates Key differences and complementarity

**Table 7:** Key differences and complementarity between Arcolezi et al. (PVLDB 2023) and this paper.

Dimension	Arcolezi et al. (PVLDB 2023)	This paper
Primary goal	Identify privacy threats and evaluate robustness of multidimensional LDP collection protocols; propose countermeasure.	Provide a compliance-oriented workflow: quantify baseline ReID risk, apply GRR-LDP, and report privacy–utility trade-offs for data sharing decisions.
Setting	Multidimensional data collection/ frequency estimation; evaluates multiple LDP protocols and attack risks.	Integrated tabular datasets in organizations; while focusing on QI-based linkage risks and operational anonymization prior to sharing.
Threat model	Explicit re-identification and attribute inference threats in multidimensional LDP; discusses risks in single and multiple collections.	Exact-match linkage attacker (QI knowledge) and baseline linkability (uniqueness, prosecutor-style risk); adds repeated-release curve for iterative sharing.
Mechanisms covered	Benchmarks several LDP mechanisms including GRR among others.	Uses GRR-LDP for categorical attributes (practical organizational pipeline), with ε sensitivity analysis.
Evaluation emphasis	Utility/robustness trade-offs for multidimensional protocols; mechanism selection guidance for practitioners.	Utility quantified both statistically (TV distance) and via a downstream ML task (accuracy/F1), tied to privacy governance and sharing scenarios.
Practical governance	Discusses protocol-level countermeasures for robustness.	Adds operational mitigations: privacy budget ledger,

To simulate a public-domain scenario, we used QIs available in open datasets (Gender, Job Title, Qualification, etc.). The same privacy and utility metrics were computed; results followed the same pattern as the primary dataset and are available upon request.

### Limitations

First, the primary evaluation uses a single employee-domain dataset; results may differ for larger, high-dimensional, sparse, or non-tabular domains. Second, privacy evaluation in this study emphasizes linkage-style proxies and risk metrics; more advanced probabilistic record linkage and inference attacks remain future work. Third, while we include a repeated-release simulation and discuss mitigations (budget ledger, memoization), deployment-grade longitudinal accounting (adaptive ε allocation, operational constraints) warrants additional investigation.

## Conclusion

This paper presents a methodology for public sector organizations to assess the risk of subject re-identification in their datasets and determine an appropriate de-identification target and level required to protect employee privacy.

By addressing the challenges associated with ReID risk, the proposed methodology provides a systematic approach to balancing privacy protection with data utility, ensuring that sensitive personal information remains secure without compromising the ability to derive meaningful insights from organizational data.

Furthermore, this paper indicates that the application of Local Differential Privacy (LDP) to integrated organizational data is an effective solution for mitigating ReID risks. The results demonstrate that differential privacy can eliminate the estimated probability of re-identification, even in datasets that are aggregated or linked across various sources. This technique not only enhances privacy protection but also ensures compliance with the Saudi Personal Data Protection Law (PDPL), meeting stringent legal requirements.

Ultimately, this research highlights the potential of differential privacy as a valuable tool for safeguarding personal data in public sector organizations, while supporting data-driven decision-making. As public sector organizations increasingly rely on integrated data for operational and strategic purposes, adopting such privacy-preserving methodologies becomes essential for maintaining trust, ensuring compliance, and protecting the privacy of employees and citizens alike. Future research can explore further enhancements to these methodologies and the scalability of differential privacy to other data categories in public sector environments.
